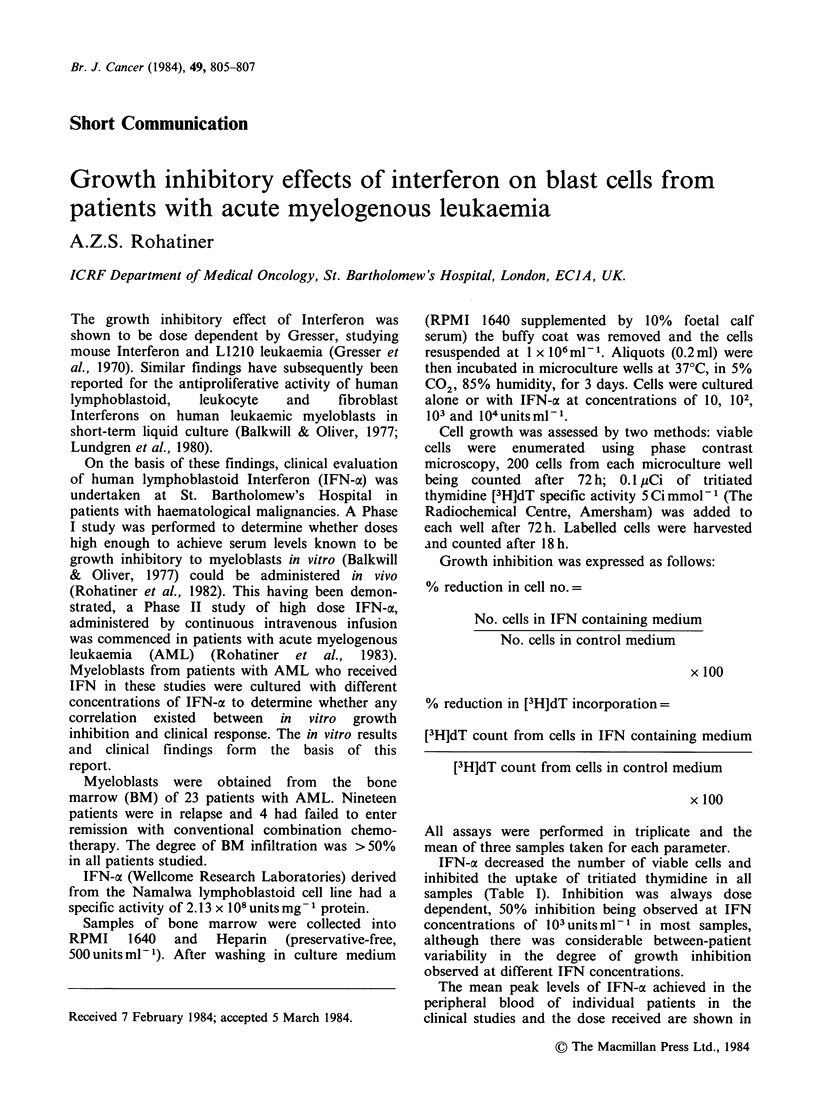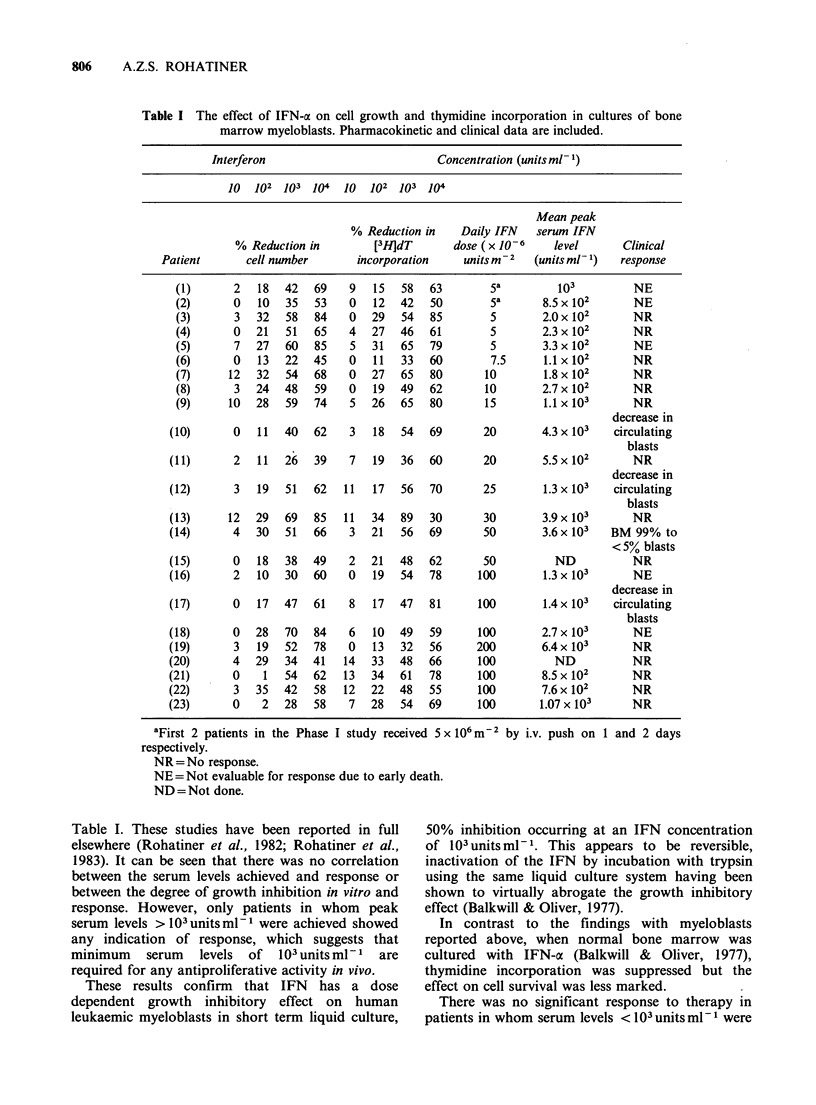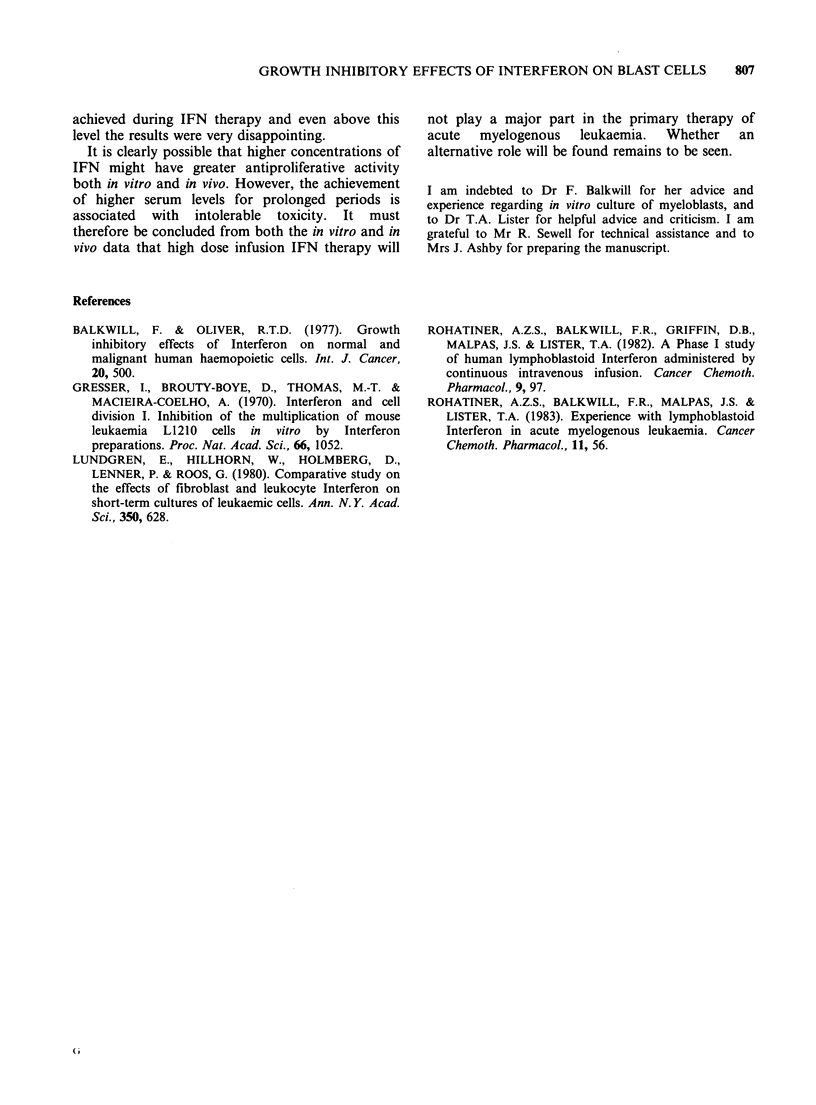# Growth inhibitory effects of interferon on blast cells from patients with acute myelogenous leukaemia.

**DOI:** 10.1038/bjc.1984.125

**Published:** 1984-06

**Authors:** A. Z. Rohatiner


*Br. J. Cancer* (1984), **49**, 805–807

### Short Communication

### Growth inhibitory effects of interferon on blast cells from patients with acute myelogenous leukaemia

A.Z.S. Rohatiner

*ICRF Department of Medical Oncology, St. Bartholomew's Hospital, London, EC1A, UK.*

The growth inhibitory effect of Interferon was shown to be dose dependent by Gresser, studying mouse Interferon and L1210 leukaemia (Gresser *et al.*, 1970). Similar findings have subsequently been reported for the antiproliferative activity of human lymphoblastoid, leukocyte and fibroblast Interferons on human leukaemic myeloblasts in short-term liquid culture (Balkwill & Oliver, 1977; Lundgren *et al.*, 1980).

On the basis of these findings, clinical evaluation of human lymphoblastoid Interferon (IFN-$\alpha$) was undertaken at St. Bartholomew's Hospital in patients with haematological malignancies. A Phase I study was performed to determine whether doses high enough to achieve serum levels known to be growth inhibitory to myeloblasts *in vitro* (Balkwill & Oliver, 1977) could be administered *in vivo* (Rohatiner *et al.*, 1982). This having been demonstrated, a Phase II study of high dose IFN-$\alpha$, administered by continuous intravenous infusion was commenced in patients with acute myelogenous leukaemia (AML) (Rohatiner *et al.*, 1983). Myeloblasts from patients with AML who received IFN in these studies were cultured with different concentrations of IFN-$\alpha$ to determine whether any correlation existed between *in vitro* growth inhibition and clinical response. The *in vitro* results and clinical findings form the basis of this report.

Myeloblasts were obtained from the bone marrow (BM) of 23 patients with AML. Nineteen patients were in relapse and 4 had failed to enter remission with conventional combination chemotherapy. The degree of BM infiltration was >50% in all patients studied.

IFN-$\alpha$ (Wellcome Research Laboratories) derived from the Namalwa lymphoblastoid cell line had a specific activity of $2.13 \times 10^8$ units mg$^{-1}$ protein.

Samples of bone marrow were collected into RPMI 1640 and Heparin (preservative-free, 500 units ml$^{-1}$). After washing in culture medium (RPMI 1640 supplemented by 10% foetal calf serum) the buffy coat was removed and the cells resuspended at $1 \times 10^6$ ml$^{-1}$. Aliquots (0.2 ml) were then incubated in microculture wells at 37°C, in 5% $CO_2$, 85% humidity, for 3 days. Cells were cultured alone or with IFN-$\alpha$ at concentrations of 10, $10^2$, $10^3$ and $10^4$ units ml$^{-1}$.

Cell growth was assessed by two methods: viable cells were enumerated using phase contrast microscopy, 200 cells from each microculture well being counted after 72 h; 0.1 $\mu$Ci of tritiated thymidine [$^3$H]dT specific activity 5 Ci mmol$^{-1}$ (The Radiochemical Centre, Amersham) was added to each well after 72 h. Labelled cells were harvested and counted after 18 h.

Growth inhibition was expressed as follows:

$$\% \text{ reduction in cell no.} = \frac{\text{No. cells in IFN containing medium}}{\text{No. cells in control medium}} \times 100$$

$$\% \text{ reduction in } [^3\text{H}]\text{dT incorporation} = \frac{[^3\text{H}]\text{dT count from cells in IFN containing medium}}{[^3\text{H}]\text{dT count from cells in control medium}} \times 100$$

All assays were performed in triplicate and the mean of three samples taken for each parameter.

IFN-$\alpha$ decreased the number of viable cells and inhibited the uptake of tritiated thymidine in all samples (Table I). Inhibition was always dose dependent, 50% inhibition being observed at IFN concentrations of $10^3$ units ml$^{-1}$ in most samples, although there was considerable between-patient variability in the degree of growth inhibition observed at different IFN concentrations.

The mean peak levels of IFN-$\alpha$ achieved in the peripheral blood of individual patients in the clinical studies and the dose received are shown in

Received 7 February 1984; accepted 5 March 1984.

© The Macmillan Press Ltd., 1984

**Table I** The effect of IFN-α on cell growth and thymidine incorporation in cultures of bone marrow myeloblasts. Pharmacokinetic and clinical data are included.

| *Interferon* | | | | | *Concentration* (*units ml*$^{-1}$) | | | | | | |
|---|---|---|---|---|---|---|---|---|---|---|---|
| | *10* | *10*$^{2}$ | *10*$^{3}$ | *10*$^{4}$ | *10* | *10*$^{2}$ | *10*$^{3}$ | *10*$^{4}$ | | | |
| *Patient* | *% Reduction in cell number* | | | | *% Reduction in* [$^{3}$*H*]*dT incorporation* | | | | *Daily IFN dose* ($\times$ *10*$^{-6}$ *units m*$^{-2}$ | *Mean peak serum IFN level* (*units ml*$^{-1}$) | *Clinical response* |
| (1) | 2 | 18 | 42 | 69 | 9 | 15 | 58 | 63 | 5[a] | $10^{3}$ | NE |
| (2) | 0 | 10 | 35 | 53 | 0 | 12 | 42 | 50 | 5[a] | $8.5 \times 10^{2}$ | NE |
| (3) | 3 | 32 | 58 | 84 | 0 | 29 | 54 | 85 | 5 | $2.0 \times 10^{2}$ | NR |
| (4) | 0 | 21 | 51 | 65 | 4 | 27 | 46 | 61 | 5 | $2.3 \times 10^{2}$ | NR |
| (5) | 7 | 27 | 60 | 85 | 5 | 31 | 65 | 79 | 5 | $3.3 \times 10^{2}$ | NE |
| (6) | 0 | 13 | 22 | 45 | 0 | 11 | 33 | 60 | 7.5 | $1.1 \times 10^{2}$ | NR |
| (7) | 12 | 32 | 54 | 68 | 0 | 27 | 65 | 80 | 10 | $1.8 \times 10^{2}$ | NR |
| (8) | 3 | 24 | 48 | 59 | 0 | 19 | 49 | 62 | 10 | $2.7 \times 10^{2}$ | NR |
| (9) | 10 | 28 | 59 | 74 | 5 | 26 | 65 | 80 | 15 | $1.1 \times 10^{3}$ | NR |
| (10) | 0 | 11 | 40 | 62 | 3 | 18 | 54 | 69 | 20 | $4.3 \times 10^{3}$ | decrease in circulating blasts |
| (11) | 2 | 11 | 26 | 39 | 7 | 19 | 36 | 60 | 20 | $5.5 \times 10^{2}$ | NR |
| (12) | 3 | 19 | 51 | 62 | 11 | 17 | 56 | 70 | 25 | $1.3 \times 10^{3}$ | decrease in circulating blasts |
| (13) | 12 | 29 | 69 | 85 | 11 | 34 | 89 | 30 | 30 | $3.9 \times 10^{3}$ | NR |
| (14) | 4 | 30 | 51 | 66 | 3 | 21 | 56 | 69 | 50 | $3.6 \times 10^{3}$ | BM 99% to <5% blasts |
| (15) | 0 | 18 | 38 | 49 | 2 | 21 | 48 | 62 | 50 | ND | NR |
| (16) | 2 | 10 | 30 | 60 | 0 | 19 | 54 | 78 | 100 | $1.3 \times 10^{3}$ | NE |
| (17) | 0 | 17 | 47 | 61 | 8 | 17 | 47 | 81 | 100 | $1.4 \times 10^{3}$ | decrease in circulating blasts |
| (18) | 0 | 28 | 70 | 84 | 6 | 10 | 49 | 59 | 100 | $2.7 \times 10^{3}$ | NE |
| (19) | 3 | 19 | 52 | 78 | 0 | 13 | 32 | 56 | 200 | $6.4 \times 10^{3}$ | NR |
| (20) | 4 | 29 | 34 | 41 | 14 | 33 | 48 | 66 | 100 | ND | NR |
| (21) | 0 | 1 | 54 | 62 | 13 | 34 | 61 | 78 | 100 | $8.5 \times 10^{2}$ | NR |
| (22) | 3 | 35 | 42 | 58 | 12 | 22 | 48 | 55 | 100 | $7.6 \times 10^{2}$ | NR |
| (23) | 0 | 2 | 28 | 58 | 7 | 28 | 54 | 69 | 100 | $1.07 \times 10^{3}$ | NR |

[a]First 2 patients in the Phase I study received $5 \times 10^{6}\,m^{-2}$ by i.v. push on 1 and 2 days respectively.
NR = No response.
NE = Not evaluable for response due to early death.
ND = Not done.

Table I. These studies have been reported in full elsewhere (Rohatiner *et al.*, 1982; Rohatiner *et al.*, 1983). It can be seen that there was no correlation between the serum levels achieved and response or between the degree of growth inhibition *in vitro* and response. However, only patients in whom peak serum levels $>10^{3}$ units ml$^{-1}$ were achieved showed any indication of response, which suggests that minimum serum levels of $10^{3}$ units ml$^{-1}$ are required for any antiproliferative activity *in vivo*.

These results confirm that IFN has a dose dependent growth inhibitory effect on human leukaemic myeloblasts in short term liquid culture, 50% inhibition occurring at an IFN concentration of $10^{3}$ units ml$^{-1}$. This appears to be reversible, inactivation of the IFN by incubation with trypsin using the same liquid culture system having been shown to virtually abrogate the growth inhibitory effect (Balkwill & Oliver, 1977).

In contrast to the findings with myeloblasts reported above, when normal bone marrow was cultured with IFN-α (Balkwill & Oliver, 1977), thymidine incorporation was suppressed but the effect on cell survival was less marked.

There was no significant response to therapy in patients in whom serum levels $<10^{3}$ units ml$^{-1}$ were achieved during IFN therapy and even above this level the results were very disappointing.

It is clearly possible that higher concentrations of IFN might have greater antiproliferative activity both *in vitro* and *in vivo*. However, the achievement of higher serum levels for prolonged periods is associated with intolerable toxicity. It must therefore be concluded from both the *in vitro* and *in vivo* data that high dose infusion IFN therapy will not play a major part in the primary therapy of acute myelogenous leukaemia. Whether an alternative role will be found remains to be seen.

I am indebted to Dr F. Balkwill for her advice and experience regarding *in vitro* culture of myeloblasts, and to Dr T.A. Lister for helpful advice and criticism. I am grateful to Mr R. Sewell for technical assistance and to Mrs J. Ashby for preparing the manuscript.

### References

BALKWILL, F. & OLIVER, R.T.D. (1977). Growth inhibitory effects of Interferon on normal and malignant human haemopoietic cells. *Int. J. Cancer*, **20**, 500.

GRESSER, I., BROUTY-BOYE, D., THOMAS, M.-T. & MACIEIRA-COELHO, A. (1970). Interferon and cell division I. Inhibition of the multiplication of mouse leukaemia L1210 cells *in vitro* by Interferon preparations. *Proc. Nat. Acad. Sci.*, **66**, 1052.

LUNDGREN, E., HILLHORN, W., HOLMBERG, D., LENNER, P. & ROOS, G. (1980). Comparative study on the effects of fibroblast and leukocyte Interferon on short-term cultures of leukaemic cells. *Ann. N.Y. Acad. Sci.*, **350**, 628.

ROHATINER, A.Z.S., BALKWILL, F.R., GRIFFIN, D.B., MALPAS, J.S. & LISTER, T.A. (1982). A Phase I study of human lymphoblastoid Interferon administered by continuous intravenous infusion. *Cancer Chemoth. Pharmacol.*, **9**, 97.

ROHATINER, A.Z.S., BALKWILL, F.R., MALPAS, J.S. & LISTER, T.A. (1983). Experience with lymphoblastoid Interferon in acute myelogenous leukaemia. *Cancer Chemoth. Pharmacol.*, **11**, 56.